# *Entamoeba* Shows Reversible Variation in Ploidy under Different Growth Conditions and between Life Cycle Phases

**DOI:** 10.1371/journal.pntd.0000281

**Published:** 2008-08-20

**Authors:** Chandrama Mukherjee, C. Graham Clark, Anuradha Lohia

**Affiliations:** 1 Department of Biochemistry, Bose Institute, Kolkata, India; 2 Department of Infectious and Tropical Diseases, London School of Hygiene and Tropical Medicine, London, United Kingdom; New York University Medical Center, United States of America

## Abstract

Under axenic growth conditions, trophozoites of *Entamoeba histolytica* contain heterogenous amounts of DNA due to the presence of both multiple nuclei and different amounts of DNA in individual nuclei. In order to establish if the DNA content and the observed heterogeneity is maintained during different growth conditions, we have compared *E. histolytica* cells growing in xenic and axenic cultures. Our results show that the nuclear DNA content of *E. histolytica* trophozoites growing in axenic cultures is at least 10 fold higher than in xenic cultures. Re-association of axenic cultures with their bacterial flora led to a reduction of DNA content to the original xenic values. Thus switching between xenic and axenic growth conditions was accompanied by significant changes in the nuclear DNA content of this parasite. Changes in DNA content during encystation-excystation were studied in the related reptilian parasite *E. invadens*. During excystation of *E. invadens* cysts, it was observed that the nuclear DNA content increased approximately 40 fold following emergence of trophozoites in axenic cultures. Based on the observed large changes in nuclear size and DNA content, and the minor differences in relative abundance of representative protein coding sequences, rDNA and tRNA sequences, it appears that gain or loss of whole genome copies may be occurring during changes in the growth conditions. Our studies demonstrate the inherent plasticity and dynamic nature of the *Entamoeba* genome in at least two species.

## Author Summary

In contrast to the perception that DNA content of an organism is stable and maintained during different conditions and life cycle stages, new evidence shows that many organisms display changes in their DNA content at different stages of their life cycle. We have earlier identified intra-cellular and inter-cellular differences in DNA content of the protist pathogen *Entamoeba histolytica* and established that this organism can tolerate large variations in DNA content during axenic culture. In this study we have made an important advancement in the understanding of amoeba biology where we have shown that changes in growth conditions and life cycle stages are accompanied by large differences in DNA content involving gain or loss of whole genome copies. This property may well regulate the outcome of infection and subsequently the disease.

## Introduction

In most eukaryotes, genomic stability is dependant on maintenance of the same genome content among individuals of the same species. This is achieved in part through the regulation of genome duplication, repair of DNA damage, and equal segregation of the duplicated genome copies. In contrast, a large number of organisms, including some plants, *Drosophila*, ciliates and other protists, and a few bacterial species, display dynamic changes in their genome or DNA content during their life cycle. Variations in DNA content between individuals of the same species may consist of duplication/deletion of large regions of chromosomes, and variable copy number of individual chromosomes or of whole genomes [1]. Endo-replication can lead to an accumulation of multiple genome copies before segregation instead of the more normal simple duplication [2].

*Entamoeba histolytica*, a protist pathogen that is the etiological agent of amoebiasis, presents an interesting example of variation in DNA content during proliferation. The life cycle of *E. histolytica* begins after the ingestion of a dormant cyst by the human host. A motile trophozoite emerges after digestion of the cyst wall during passage through the small intestine and colonises the large intestine, where it proliferates while feeding on the resident bacterial flora. To complete the life cycle, trophozoites develop into quadri-nucleate cysts and pass out into the external environment with the host faeces. *E. histolytica* may exist as a commensal in the human intestine, or cause dysentery or extra-intestinal abscesses. Proliferation of trophozoites in the intestinal lumen occurs in a xenic environment where they co-exist with different anaerobic and micro-aerophilic microbial species. In contrast, during invasion of the intestinal epithelium or extra-intestinal sites such as liver abscesses, amoeba cells proliferate in an environment free of other microbes.

In early studies, cultivation of *E. histolytica* outside the host required a microbial flora [3] and conversion of *E. histolytica* trophozoites into mature cysts has still not been accomplished *in vitro* in the absence of associated bacteria [4]. Axenic cultivation of *E. histolytica* became possible in 1961 and study of its biology *in vitro* greatly expanded when the medium TYI-S-33 was developed [5]. Almost all cellular and molecular biological studies now use axenically cultured *E. histolytica*; indeed sequencing of its genome [6],[7] would not have been possible without axenic cultures.

Although the tetra-nucleated *E. histolytica* cyst must contain at least one to two genome copies (1n–2n) in each of the nuclei, the ploidy of proliferating cells is not known. Analysis of the *E. histolytica* karyotype indicated the presence of at least 4 functional copies of many structural genes in different isolates [8], and therefore probably a ploidy that is a multiple of four.

We have sought to understand the mechanisms that regulate proliferation of this parasite [9],[10]. Our previous studies have shown that axenic cultures of *E. histolytica* trophozoites contain heterogeneous populations of cells with 1, 2 or more nuclei [10]. Additionally, the DNA content of individual nuclei may vary six-fold [10]. Similar to *E. histolytica*, variation of cellular DNA content in trophozoites during axenic cultivation has been observed in *E. invadens* [11],[12]. This heterogeneity may occur due to the absence of mechanisms known to coordinate DNA replication, mitosis and cell division in model organisms such as yeasts and vertebrates [13],[14],[15] or may be due to inherent plasticity as observed in numerous organisms with dynamic genomes. In any case, diversity in DNA content amongst a population of cells in axenic cultures is commonly observed in *E. histolytica*.

Following on from our earlier studies on *E. histolytica* HM-1:IMSS, in this study we have examined the DNA content of additional isolates of *E. histolytica* in axenic culture. We have also examined the heterogeneity and DNA content of *E. histolytica* trophozoites growing under different conditions - namely with associated microbes (xenic) and without any associated organisms (axenic). The related species *E. invadens* has been used to analyse changes in DNA content following excystation. Our results show that endo-replication of DNA occurs both when *E. histolytica* cells are shifted from xenic to axenic growth and when *E. invadens* cysts release trophozoites, and that this change is reversible.

## Materials and Methods

### Cell culture and maintenance

The axenic isolates used in this study include the standard laboratory strains *E. histolytica* HM-1:IMSS and *E. histolytica* 200:NIH, and *E. histolytica* KCG:0687:3. The latter was axenised in 1987 [16]. Trophozoites were maintained axenically in TYI-S-33 medium [5]. Clinical isolates *E. histolytica* DS4-868 and 2592100 were isolated from patients with diarrhea [17] and were grown under xenic conditions in LYSGM (http://homepages.lshtm.ac.uk/entamoeba/labman.htm) supplemented with 5% adult bovine serum and rice starch. Axenic cultures were derived from these xenic isolates by the classical method of Diamond and grown in LYI-S-2 with 15% adult bovine serum [18]. For re-xenization, the axenic cells were re-associated with the bacterial flora obtained from the original xenic cultures, and grown in LYSGM as above [18].

*E. invadens* IP-1 cells were maintained axenically in TYI-S-33 medium at room temperature (25°C) and sub-cultured every two weeks. Encystation of trophozoites was carried out as described in Sanchez *et al.* 1994 [19]. Excystation took place in TYI-S-33 medium after washing the cysts with 0.1% Sarkosyl to eliminate trophozoites. Conversion of trophozoites to cysts was monitored by staining with 25 µM Calcofluor or survival in 0.1% Sarkosyl [20]. Cysts and trophozoites were fixed 7 days after induction of encystation or excystation for measurement of DNA content.

### Counting cells and nuclei

Cells were counted in a haemocytometer for determining cell number. Number of nuclei per cell were analysed using DAPI or hematoxylin stained amoeba cells under a fluorescence or bright field microscope (Zeiss Axiovert 200 M, Axiovision v 4.6).

### Analysis of whole cell DNA content by flow cytometry

Log phase cells were harvested 48 h after inoculation, fixed in 70% ethanol and kept at −20°C for 2 h. Fixed cells were washed with 1× PBS, treated with RNase A (0.2 mg/ml, Sigma, USA) for at least 6 h at room temperature and then stained with propidium iodide (PI) (0.1 mg/ml, Sigma, USA) for 10 min in the dark at room temperature. Washed cells were passed through a 40 µm nylon mesh (Becton Dickinson, USA) to eliminate aggregates and debris before acquiring the data in a flow cytometer (FACSCalibur, Becton Dickinson, USA). For measurement of PI fluorescence (DNA content), cells were excited with 488 nm light and emission was measured through a 575 DF20 filter. Data from 10,000 cells were recorded for each experiment and analyzed using CELLQUEST software (Becton Dickinson, USA).

### Analysis of cell division by CFSE and flow cytometry

48 h grown log phase HM-1:IMSS cells were synchronized partially by changing the growth medium every 24 h for 3 days (10). These cells were harvested and 10^7^ cells/ml were labelled with 5 µM carboxyfluorescein diacetate succinamidyl ester (CFSE; Invitrogen, USA) for 5 min at room temperature. CFSE labeled and unlabelled cells were inoculated in growth medium and harvested at 12 h intervals up to 72 h after inoculation. Cells were fixed in 70% ethanol, washed in 1× PBS and analysed by flow cytometry as described above. Auto-fluorescence was determined from unstained fixed cells harvested at the time of CFSE staining. CFSE fluorescence was estimated in cells excited with 488 nm light and emission was measured with 525 DF20 filter. Data from 10,000 cells were recorded for each experiment and analyzed using CELLQUEST software (Becton Dickinson). Since PI and CFSE have overlapping emission spectra, cellular DNA content was estimated using a parallel culture without CFSE staining.

### Scanning cytometry to determine the nuclear DNA content and area

Ethanol fixed cells were stained with DAPI (0.1 µg/ml, Sigma) for 10 min, washed once with 1× PBS and then spread on glass slides. Each slide was scanned for DNA content of individual nuclei under a 40× objective of a Zeiss Axiovert 200 M fluorescence microscope fitted with the MetaCyte scanning cytometer and Metafer 4 software (Zeiss, Germany). A minimum of 2000 nuclei was scanned for each axenic isolate, while for the xenic isolates a minimum of 500 nuclei was scanned for measurement of nuclear DNA content and nuclear contour area. Both the DAPI fluorescence values and nuclear contour area (x-axis) were represented as histograms. The scan yields varying numbers of nuclei with different fluorescence values. Number of nuclei on the y-axis were represented as a fraction of the highest number of nuclei obtained in any one sub-class of each scan.

### Immuno-histochemistry of intestinal sections

Paraffin embedded intestinal tissue sections (a kind gift from John Williams, Diagnostic Parasitology Laboratory, LSHTM) were de-paraffinised using xylene, 3× for 5 min each, followed by ethanol in decreasing concentrations (100%, 2×, 5 min; 95%, 2×, 5 min; 80%, 1×, 5 min; 50%, 1×, 5 min). De-paraffinised tissue sections were blocked with 2.5% horse serum for 1 h at room temperature, followed by incubation with anti-Eh lectin (heavy subunit) rabbit polyclonal antibody (1∶500) for 1 h at room temperature (kind gift from Prof. Bill Petri, University of Virginia). After several washes in 1× PBS, ImmPress reagent (anti-rabbit antibody with conjugated alkaline phosphatase; Vector laboratories, USA) was added for 30 min at room temperature. Colour development was carried out according to the manufacturer's instructions (Vector Laboratories, USA). Nuclei were stained with Hematoxylin (Delafield) stain solution (Merck, Germany).

### Genomic DNA isolation and quantitative PCR

Xenic cultures were harvested at 275× g for 5 min. The supernatant containing bacterial flora was decanted, the amoeba cell pellet washed in 1× PBS and then fixed with 70% ethanol at −20°C for 2 h at least. The fixed cells were washed with 1× PBS and passed through a nylon mesh (40 µm) to remove large particles and debris. Axenic cultures were harvested and the cell pellet was fixed with 70% ethanol at −20°C for 2 h at least. Genomic DNA was isolated from approximately 1×10^2^ fixed cells of 2592100 from xenic and axenic cultures as well as cysts and trophozoites of *E. invadens* IP-1 using a Qiaamp DNA Micro kit (Qiagen, Germany). To compare the abundance of different target sequences between *E. histolytica* axenic/xenic cells and *E. invadens* trophozoites/cysts we identified a representative set of protein coding sequences distributed on independent contigs, ribosomal DNA and tRNA sequences. Specific primer pair sequences, contig numbers for *E. histolytica* and locus numbers for *E. invadens* are shown in Tables S1 and S2. qPCR analysis was carried out in a 7500 SDS Real-time instrument (Applied Biosystems, USA) using SYBR GREEN PCR Master Mix (Applied Biosystems, USA) according to the manufacturer's instructions. *Eh Actin* was used as a control for *E. histolytica* whereas *Ei β-Tubulin* was the control for *E. invadens* sequences. All reactions were carried out in triplicate and average threshold cycle values were calculated to determine the ratio of control gene to target gene (F = C_t_ control gene/C_t_ target gene). Semi-quantitative PCR was carried out with tRNA primers (described in Table S1). In case of semi-quantitative PCR, relative intensity (F) of tRNA PCR products were measured densitometrically (normalised against PCR products of control sequences e.g. *Eh actin* and *Ei β tubulin*). The ratio of the F values of a target sequence in *Eh* axenic vs xenic or *Ei* trophozoite vs cyst was used to determine its relative abundance. Control experiments with three primer sets using axenic DNA with added microbial DNA, to identify the effect of bacteria on PCR reactions using xenic DNA, did not show any significant differences from axenic DNA alone.

### Statistical analysis

Mean and standard deviation values of nuclei per cell from different isolates were calculated using Kyplot. *p*-values of pair-wise comparison (two-sided) of axenic and xenic cultures was performed using t-test.

## Results

### Different isolates of *Entamoeba histolytica* display similar heterogeneity in DNA content

*E. histolytica* HM-1:IMSS was first axenised from a Mexican patient over 30 years ago [6] and since then it has been the preferred strain for study, including genome sequencing [7]. Studies on the regulation of proliferation or cell division cycle of HM-1:IMSS have identified two unique characteristics of proliferation in *E. histolytica*. Firstly, unlike most eukaryotes, the cell division cycle of *E. histolytica* lacks control mechanisms that ensure alternation of DNA duplication and mitosis [13],[15],[21]. Secondly, axenic cultures of *E. histolytica* HM-1:IMSS contain cells with multiple nuclei and the DNA content of individual nuclei may vary dramatically [10]. This heterogeneity does not affect the phenotypic properties or stability of the organism upon long-term culture.

**Figure 1:**
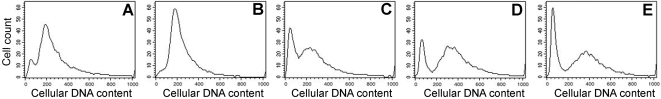
Heterogeneity in the distribution of average DNA content is similar in different axenic isolates of *Entamoeba histolytica*. Cells from *E. histolytica* isolates (A) HM-1:IMSS, (B) 200:NIH, (C) KCG:0687:03, (D) 2592100 and (E) DS4-868 were fixed 48 h after sub-culture, stained with PI and the cellular DNA content (x-axis) was analyzed by flow cytometry. The y-axis represents cell count.

In order to determine whether other *E. histolytica* isolates exhibited variation in their DNA content, we compared four other *E. histolytica* isolates with HM-1:IMSS (Fig. 1). Analysis of log phase cultures showed that all the *E. histolytica* isolates studied contained cells with heterogeneous amounts of DNA (Fig. 1). Discrete populations of cells with 1n and 2n DNA content alone were not seen in any of the isolates. The histograms showed a broad distribution suggesting the presence of cells with widely varying DNA contents [9],[22]. Thus, heterogeneity in total DNA content among individual cells appears to be a common property of *E. histolytica* trophozoites in axenic culture and is not limited to HM-1:IMSS.

**Figure 2:**
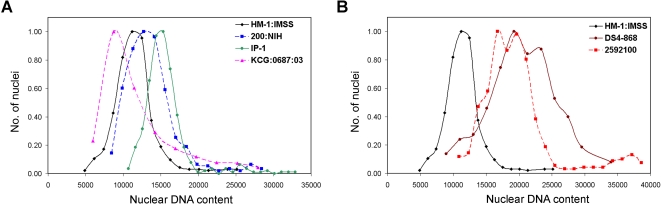
Distribution of nuclear DNA content in different axenic isolates of *E. histolytica*. Cells from 48 h cultures of (A) *E. histolytica* HM-1:IMSS; 200:NIH; KCG:0687:03 and *E. invadens* IP-1 and (B) *E. histolytica* HM-1:IMSS, 2592100 and DS4-868 were fixed and stained with DAPI to analyse the DNA content of individual nuclei. The DAPI fluorescence values for each isolate were drawn as individual histograms. The x-axis shows fluorescence values, representing the nuclear DNA content on a linear scale and the y-axis represents the number of nuclei as a fraction of the highest number of nuclei obtained in any sub-class of each scan.

The cellular DNA content profile includes cells with different numbers of nuclei. Therefore, we analysed the DNA content of individual nuclei in each of the isolates to establish whether the heterogeneity seen earlier for HM-1:IMSS [10] was present. All axenic *E. histolytica* isolates displayed similar heterogeneity in the distribution of nuclear DNA content (Fig. 2 A–B). However, the average DNA contents of DS4-868 and 2592100 nuclei were greater than for the other *E. histolytica* isolates (Fig. 2B). DS4-868 and 2592100 were axenised recently compared to the other isolates, which have been in axenic culture for decades. It is possible that the higher DNA content of the recent axenisations will decrease over time.

Taken together, the data presented in Figs. 1 and 2 show that both cellular and nuclear DNA contents vary amongst cells in all axenic *E. histolytica* isolates studied. Since *E. histolytica* cells normally proliferate in the xenic environment of the large intestine, we compared the DNA content of two isolates under both xenic and axenic conditions.

### The average DNA content of *E. histolytica* trophozoites is much less in xenic culture than in axenic culture

**Figure 3:**
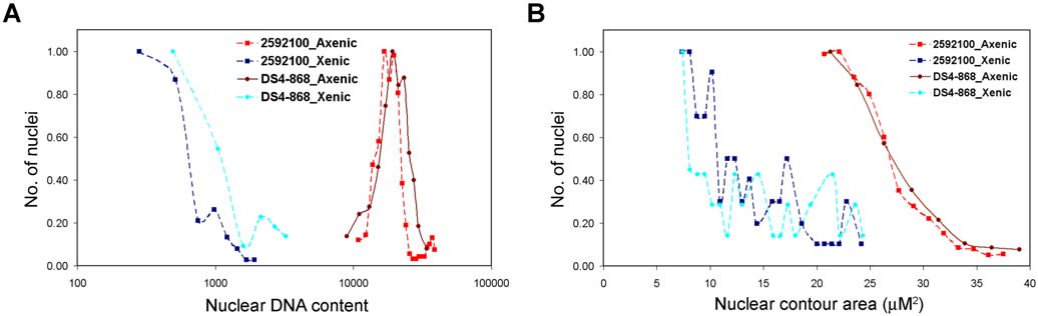
Nuclear DNA content of *E. histolytica* trophozoites is higher during axenic growth. (A) Cells from xenic and axenic cultures of 2592100 and DS4-868 were fixed and stained with DAPI to analyze the DNA content of individual nuclei. The x-axis is fluorescence plotted on a logarithmic scale to demonstrate the great difference in DNA content between xenic and axenic nuclei. The y-axis is as in fig. 2. (B) Nuclear contour area was measured as an indicator of differences in nuclear size or volume in cells growing under xenic and axenic conditions. The y-axis is as above.

The original xenic cultures of *E. histolytica* HM-1:IMSS, 200:NIH and KCG:0687:3 are no longer available. *E. histolytica* 2592100 and DS4-868 were isolated recently and are maintained as both xenic and axenic cultures. A comparison of these paired cultures showed differences in cellular and nuclear size as well as in DNA content. The nuclear DNA content of both *E. histolytica* isolates was at least 10 fold lower when grown under xenic conditions compared to axenic cultures (Fig. 3A). Since axenic cultures of *E. histolytica* are derived from xenic cultures by step-wise elimination of associated microbes, it is likely that during this adaptation the *E. histolytica* cells increased in size (data not shown) and accumulated large amounts of DNA. Increased nuclear DNA content is possible if cells undergo endo-replication of the genome without mitosis. Such an event leads to accumulation of multiple copies of the genome (polyploidy) and is likely to be accompanied by an increase in nuclear size. Analysis of nuclei in *E. histolytica* 2592100 and DS4-868 xenic and axenic cultures showed a size increase in axenic cultures commensurate with the increase in DNA content (Fig. 3B). Furthermore, analysis of the number of nuclei in each cell showed that, in both isolates, multi-nucleated cells were more frequent (12–15%) in axenic cultures (Table 1). Thus *E. histolytica* cells contained increased amounts of nuclear DNA and cultures contained more multi-nucleate cells after adapting to axenic growth.

**Table 1:** Percentage of uni-nucleated, bi-nucleated and multi-nucleated cells in different isolates of *E. histolytica*.

Isolate	Percentage of nuclei±S.D. (n = 3)		
	1	2	>2
HM-1:IMSS	87.33±1.15	11.33±1.15	1.67±0.58
200:NIH	87.67±1.15	11.33±1.53	1.33±0.58
KCG:0687:03	87.33±2.08	11.00±1.00	1.67±0.58
2592100 (Axenic)	85.33±1.53**	13.17±1.04*	1.50±0.50^N.S.^
2592100 (Xenic)	97.33±0.58**	2.33±1.15*	0.70±0.10^N.S.^
DS4-868 (Axenic)	83.67±1.53**	14.67±2.08**	1.67±0.58^N.S.^
DS4-868 (Xenic)	96.33±1.53**	2.73±0.55**	0.63±0.15^N.S.^

Axenic and xenic cells were stained with DAPI and the numbers of nuclei were counted in each cell. At least 100 (xenic) and 500 (axenic) cells were counted in three replicates to determine the percentage of uni-nucleated, bi-nucleated and multi-nucleated (>2) cells. *p*< = 0.05 is considered to be significant. ^*^ and ^**^ denote *p*< = 0.05 and *p*< = 0.01 respectively. N.S. = not significant (*p*> = 0.05). *p* value was obtained after comparison between xenic and axenic growth condition of 2592100 and DS4-868 respectively.

**Figure 4:**
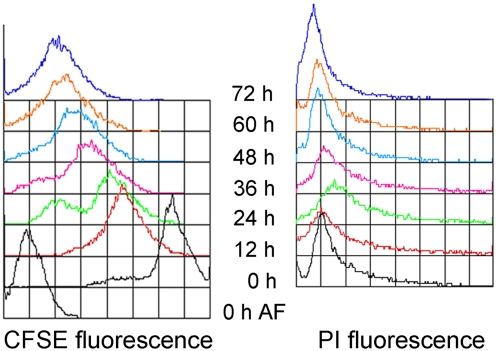
Cell division of *E. histolytica* in axenic cultures. CFSE labeled *E. histolytica* HM-1:IMSS cells were monitored for cell division and proliferation over time. CFSE labeled cells were harvested and fixed at 12 h intervals. CFSE fluorescence of these samples is plotted as an overlay diagram. PI fluorescence (cellular DNA content) from a parallel culture is also plotted as an overlay. Auto-fluorescence (AF) was determined at 0 h. y-axis represents cell count. The data is representative from 3 independent replicates.

In order to determine if the polyploid or multi-nucleate axenic cells were proliferating cells, we labeled axenic cells with CFSE which is segregated into daughter cells so that the individual cell's fluorescence is reduced with each cell division. These cells were harvested every 12 h for 3 days and the amount of CFSE was monitored along with cellular DNA content (Fig. 4). Fig. 4 shows that the CFSE fluorescence diminished progressively over time, while the DNA content profile displayed characteristic heterogeneity (Fig. 4). A proportionate increase in cell numbers (Table 2) showed that cell division occurred consistently. Multi-nucleated cells were distributed in similar numbers throughout the time course (Table 2). If polyploid or multi-nucleated cells were non-dividing, these would have persisted as a subpopulation with high CFSE and perhaps increasing propidium iodide values over the time course.

**Table 2:** Cell number and percentage of uni-nucleated, bi-nucleated and multi-nucleated cells during cell division of *E. histolytica* in axenic cultures.

Time (h)	(No. of cells±S.D.)×10^4^/ml (n = 3)	Percentage of nuclei per cell±S.D. (n = 3)		
		1	2	>2
0	6.0±0.0	84.7±0.8	12.5±0.5	2.5±0.5
12	10.5±0.5	86.4±0.4	11.5±1.5	1.9±0.9
24	17.5±1	90.9±1.2	7.5±0.5	1.2±0.2
36	29.6±0.9	87.0±1.0	11.6±0.6	1.6±0.6
48	56.2±2.2	88.3±1.3	9.4±0.4	1.8±0.8
60	90.5±4.5	89.5±0.5	8.7±0.3	1.7±0.7
72	200.1±9.4	89.1±1.1	9.3±0.7	1.8±0.2

Cell numbers were counted for the CFSE labeled and unlabelled cultures at the indicated time points. The number of nuclei in each cell was counted manually in at least 500 cells after staining with DAPI, and the percentage of uni-nucleate, bi-nucleate and multi-nucleated cells was calculated.

**Figure 5:**
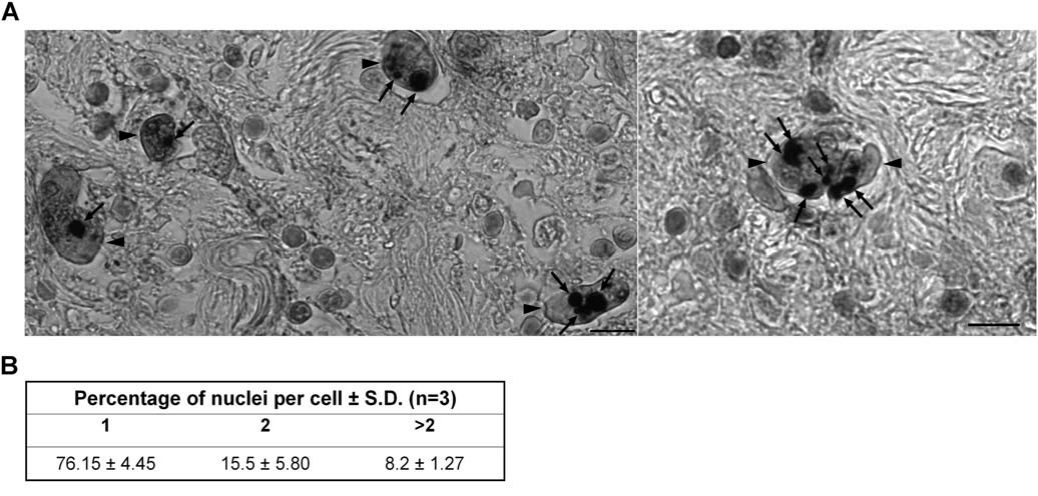
*E. histolytica* trophozoites show heterogeneity in nuclear number during intestinal tissue invasion. Intestinal tissue sections were stained with polyclonal anti-Eh lectin antibody to identify amoeba cells followed by hematoxylin staining of the nuclei. (A) Two representative images are shown where amoeba cells (indicated by arrowheads) contain different number of nuclei (indicated by arrows). Scale bar is 10 µm. (The image of each amoeba cell was verified at high magnification to be of a single intact cell and the size of host cell nuclei was found to be double that of the amoeba nuclei- data not shown). (B) Intestinal tissue sections (n = 3) were counted for the presence of uni- (1), bi- (2) and multi- (>2) nucleated amoeba cells.

We next examined if *E. histolytica* cells showed similar heterogeneity during infection of the human host and tissue invasion. We stained human intestinal tissue sections from a case of amoebic colitis (Fig. 5A) and visually scored 296 amoebae for multi-nucleation (Fig. 5B). Our data showed that only 75% of the amoebae were uni-nucleated in the intestinal sections. The increased number of multi-nucleated amoebae in intestinal tissue sections is similar to amoebae growing under axenic conditions as shown above. Although the possibility of ingestion of host cell nuclei by some of the amoebae that appear to be multi-nucleated cannot be ruled out, it should be noted that the average size of host cell nuclei was at least two-fold larger (data not shown) than the amoeba nuclei and therefore did not affect our analysis of nuclear number estimation in tissue amoebae. However, estimation of intestinal amoeba nuclear size showed similarity with the size of nuclei in xenic cultures (data not shown). Taken together the data show that heterogeneity of nuclear DNA content and nuclear number appears to be an inherent property of *Entamoeba* irrespective of *in vitro* growth conditions and also reflects an *in vivo* phenomenon.

### Re-xenisation of axenically growing *E. histolytica* leads to a reduction in nuclear DNA content

**Figure 6:**
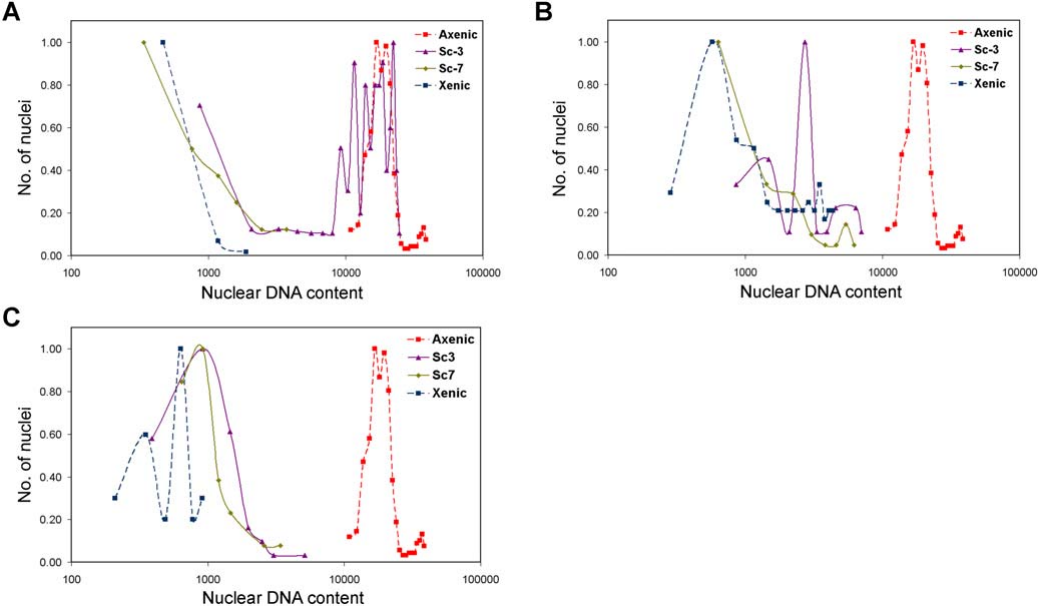
Re-xenisation leads to reduction in DNA content of axenic trophozoites. Axenic cultures of *E. histolytica* 2592100 were re-xenized by inoculation into xenic culture medium containing the original bacterial flora. Cells were fixed for analysis of nuclear DNA content after the third (Sc3) and seventh (Sc7) subcultures. DNA content (x-axis) of re-xenized samples as well as the xenic and axenic cells of the same isolate were analyzed by scanning cytometry and are represented as histograms. y-axis is described above. The three panels A B and C depict independent re-xenisation experiments.

We then tested whether the increase in DNA content in axenic trophozoites was a terminal event or if it reverted to its original values following re-association with bacteria. Re-xenisation was carried out using isolate 2592100 and cells were fixed at different time-points after addition of axenic cells to xenic culture medium containing its original bacterial flora. During re-xenisation, the nuclear DNA content of axenic cells decreased gradually. Once adaptation to xenic growth was complete the nuclear DNA content has been reduced by 10–20 fold and was comparable to that in the original xenic cells (Fig. 6 A–C). This reduction in nuclear DNA content was observed in three independent replicates. Similar results were obtained for DS4-868 (not shown). Nuclear size also showed a similar reversion (data not shown). While cultures took approximately 3 weeks to return to stable xenic levels of growth, the changes in nuclear DNA content occurred very soon after transfer to xenic growth medium with the microbial flora. The rate of change appeared to be dependant on the growth of the amoeba cells. Although identical culture conditions were maintained for the three independent replicates, the cells in Fig. 6C adapted more quickly and grew faster than those in Figs. 6A and B. A reduction in nuclear DNA content was already detectable after the first sub-culture (not shown).

### The nuclear DNA content of *Entamoeba invadens* cysts and trophozoites

The reptilian parasite *Entamoeba invadens* is a useful model system for analysis of DNA content during differentiation from cyst to trophozoite and back, since this conversion cannot be achieved under axenic conditions in *E. histolytica*. *E. invadens* IP-1 trophozoites growing axenically were induced to form cysts which were then purified (as described in the methods section). Our earlier report [12] compared the total trophozoite and cyst DNA content by flow cytometry, where both populations were heterogenous and therefore the estimates of DNA content are not comparable with the current study.

**Figure 7:**
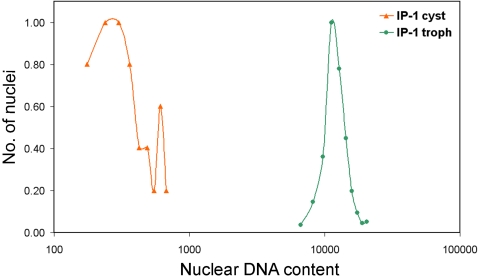
*E. invadens* IP-1 trophozoites contain 40 fold higher nuclear DNA content than cysts. The DAPI fluorescence values of *E. invadens* cyst and trophozoite nuclei are compared on a logarithmic scale (x-axis). y-axis represents the number of nuclei as a fraction of the highest number of nuclei obtained in any one sub-class of each scan.

In this study, cysts were fixed for nuclear DNA content estimation and also used for excystation in axenic medium. Comparison of the nuclear DNA content in cysts and excysted trophozoites showed that trophozoite nuclei contained almost 40 fold more DNA than each cyst nucleus (Fig. 7). Interestingly, the amount of DNA in each cyst nucleus was not identical but showed a broad distribution which suggests that the cyst nuclei may contain more than 1n or 2n DNA content. Furthermore the nuclear DNA content of excysted trophozoites returned within one week to a level similar to that of trophozoites maintained in axenic culture (data not shown). We conclude therefore that both *E. histolytica* and *E. invadens* demonstrate amplification and reduction of nuclear DNA content under different growth conditions and life cycle phases.

### Endo-replication of the entire *Entamoeba* genome occurs during axenisation and excystation

**Figure 8:**
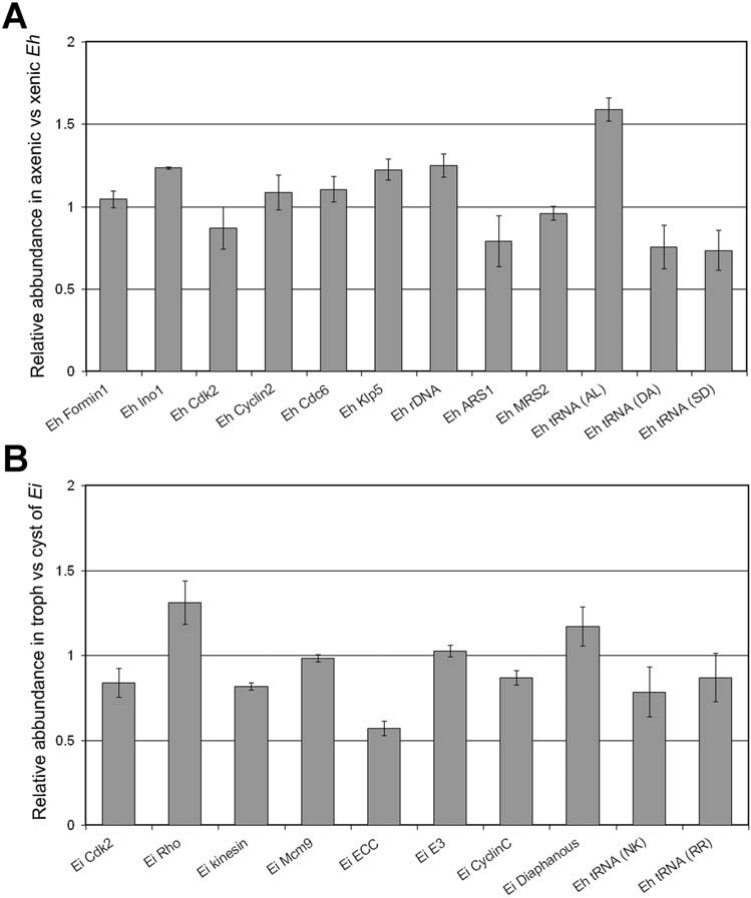
Relative abundance of a representative set of target sequences in *E. histolytica* and *E. invadens*. (A) qPCR (protein coding sequences and rDNA) and semi-quantative PCR (tRNA genes) was performed on genomic DNA isolated from fixed cells of xenic and axenic *E. histolytica* 2592100 using specific primer sets described in Table S1. Relative abundance of target sequences was calculated as described in the methods section. Values greater than 1 on the y-axis indicate greater abundance in axenic cells and values less than 1 on the y-axis indicate greater abundance in xenic cells. (B) qPCR (protein coding sequences) and semi-qPCR (tRNA genes) was performed on genomic DNA isolated from *E. invadens* IP-1 trophozoites and cysts using specific primer sets described in Table S2. Relative abundance was calculated as described in the methods section. Values greater than 1 on the y-axis indicate greater abundance in trophozoites and values less than 1 on the y-axis indicate greater abundance in cysts.

Axenically cultured trophozoites of *E. histolytica* showed 10–20 fold excess DNA compared to their xenic counterparts. Similarly *E. invadens* trophozoites upon excystation from cysts showed a 40 fold amplification of nuclear DNA. In order to determine whether the increased nuclear DNA content was due to expansion of the whole genome or only specific regions of the genome, we compared the copy numbers of protein coding sequences from different contigs, tRNA genes and ribosomal DNA in axenic and xenic DNA from *E. histolytica*. Similarly, a different set of target sequences was examined in cyst and trophozoite DNA of *E. invadens* (Tables S1, S2). The data are presented as relative abundance of the target sequence in *E. histolytica* axenic cells compared to xenic cells (Fig. 8A) and in *E. invadens* trophozoites compared to cysts (Fig. 8B). Our data show that the relative abundance of the different target sequences varied by 10–20% between *E. histolytica* axenic and xenic cells without any specific bias. Of the tRNA sequences that were tested only one (from array [AL]) showed an increase of 50% in axenic cells compared to xenic cells, while three other tRNA sequences showed a 20% decrease. A similar profile was observed for most of the targets in *E. invadens* except for the Encystation Complex Component (ECC) which showed 50% decrease in relative abundance in trophozoites compared to cysts.

Along with chromosomal DNA, the *E. histolytica* nucleus contains episomal 25 kb ribosomal DNA circles in approximately 200 copies [23]. In order to ascertain whether the copy number of rDNA circles might be contributing to the difference in nuclear DNA content, we calculated the ratio of chromosomal genes to rDNA in xenic and axenic cultures. Fig. 8A shows that, in *E. histolytica* 2592100, the rDNA content relative to *Eh actin* appeared to be slightly lower during xenic growth compared to axenic growth. The rDNA circles form a substantial component (ca 20%) of the nuclear DNA content of axenic cells but the modest differences in rDNA copy number would not be enough to account for the large variation in total DNA content or nuclear size observed. Overall our data show relatively small fluctuations in the copy number of randomly selected target sequences, and do not support the expansion of specific DNA regions as being responsible for the total increase in nuclear DNA of axenic compared to xenic cells. A whole genome analysis may identify certain preferential sites of expansion. However, the current analyses suggest that the increase in nuclear DNA content and nuclear size during axenisation or following excystation must involve an increase of most if not all the nuclear DNA.

## Discussion

This study highlights several important properties of the protozoan parasites *E. histolytica* and *E. invadens*. Firstly, during the change of growth conditions from xenic to axenic culture, *E. histolytica* undergoes increases in nuclear size and DNA content. This phenomenon is reversible when axenic cultures are re-associated with a bacterial flora. In the social amoeba *Dictyostelium discoideum*, it was demonstrated that amoebae grown in axenic culture tended to be multi-nucleate and had a greater DNA content per nucleus than amoebae grown on a bacterial substrate, which were uni-nucleate [24]. Wild type cells grew xenically while axenic growth was favoured in phagocytosis and motility deficient mutants of *Dictyostelium* [25]. These mutants have also been associated with defects in cytokinesis and increased multi-nuclearity during axenic growth [26]. Although loss of microbial substrates appears to be linked with increased DNA content during axenic growth in both *D. discoideum* and *E. histolytica* it may not be the only factor involved since an increase in nuclear DNA content of 40–50 fold is also observed when *E. invadens* trophozoites emerge from cysts into axenic cultures. This increase is also accompanied by nuclear size expansion (data not shown).

Increase in genome content and cell size is often associated with increased metabolic activity [2]. It is possible that when *E. histolytica* cells adapt to axenic growth, the absence of certain nutrients normally derived from bacteria may enforce an increase in gene expression to compensate, which may in turn require an increase in gene copy number that is achieved through endo-replication of genome content. Therefore identification of genes involved in the adaptation from xenic to axenic growth and the increase in DNA content are of immediate interest. Comparative transcriptional profiling of gene expression in xenic and axenic cultures of *E. histolytica* [27] showed significant changes in the expression of several hypothetical proteins and cyst-specific proteins, possibly due to spontaneous encystation occurring in xenic culture [27]. Whether these proteins or networks that regulate gene expression during encystation play a role in inducing endo-replication is not yet known. Changes in the expression levels of known homologs of cell cycle regulating genes were not detected [27]. Apart from regulation of gene expression, epigenetic mechanisms, such as DNA methylation or histone modification, may also regulate the inter-conversion between trophozoites and cysts and the DNA content of the organism. Indeed, short chain fatty acids, which are known inhibitors of histone deacetylase, have been shown to restrict the ploidy of *E. invadens* in axenic cultures [11].

Our data suggest that endo-replication leads to polyploidy in axenic trophozoites. Cells with polyploid nuclei are of necessity the normal precursors of quadri-nucleate cysts, and the successful conversion of polyploid trophozoites to cysts in *E. invadens* suggests that these cells are indeed able to fill this role, at least in this species. Our data show that even after tissue invasion *E. histolytica* trophozoites show multiple nuclei and heterogeneity in nuclear size reflecting the similarity between the *in vivo* and *in vitro* conditions. Parasites that have invaded host tissues do not encyst and cannot complete the life cycle. It is likely that unknown environmental factors are responsible for the lack of encystation in tissue rather than an inherent inability of these parasites to complete the life cycle.

Re-xenisation of *E. histolytica* isolates leads to a reduction of nuclear DNA content, confirming that endo-replication is a dynamic change and that axenic cells are not terminal cells, except perhaps if they have been maintained in axenic medium over extended periods as seen in HM-1:IMSS [28]. Since the difference in DNA content between *E. invadens* trophozoite and cyst nuclei is around 40 fold, only one or a few genome copies are sequestered in each of the cyst nuclei while the additional copies must be lost. Reduction of DNA content while switching back from axenic to xenic conditions suggests that a mechanism for genome copy number reduction also exists in *E. histolytica*. This may include multiple nuclear and cell division cycles in response to re-xenisation. It is also possible that specific regions of the genome are differentially amplified or deleted – such as repetitive heterochromatin. Selective loss of heterochromatin during development has been shown in various cell types [1]. Almost 47% of the amoeba genome is non-coding and its repetitive heterochromatin is not well understood. However, based on the modest changes in copy number of randomly selected DNA sequences, coupled to the observed changes in nuclear size and DNA content, the most plausible mechanism may be gain and loss of whole genome copies. In conclusion our studies demonstrate the inherent plasticity and dynamic nature of the *E. histolytica* genome, and suggest that the observations made *in vitro* reflect the natural situation *in vivo*.

## Supporting Information

Table S1 (0.05 MB DOC)

Table S2 (0.04 MB DOC)

## References

[1] Parfrey LW, Lahr DJ, Katz LA (2008). The dynamic nature of eukaryotic genomes.. Mol Biol Evol.

[2] Edgar BA, Orr-Weaver TL (2001). Endoreplication cell cycles: more for less.. Cell.

[3] Cleveland LR, Sanders EP (1930). The production of bacteria free amebic abcesses in the livers of cats and observations of the amebae in various media with and without bacteria.. Science.

[4] Eichinger D (2001). Encystation in parasitic protozoa.. Curr Opin Microbiol.

[5] Diamond LS, Harlow DR, Cunnick CC (1978). A new medium for the axenic cultivation of *Entamoeba histolytica* and other Entamoeba.. Trans R Soc Trop Med Hyg.

[6] Clark CG, Alsmark UC, Tazreiter M, Saito-Nakano Y, Ali V (2007). Structure and content of the *Entamoeba histolytica* genome.. Adv Parasitol.

[7] Loftus B, Anderson I, Davies R, Alsmark UC, Samuelson J (2005). The genome of the protist parasite *Entamoeba histolytica*.. Nature.

[8] Willhoeft U, Tannich E (1999). The electrophoretic karyotype of *Entamoeba histolytica*.. Mol Biochem Parasitol.

[9] Gangopadhyay SS, Ray SS, Kennady K, Pande G, Lohia A (1997). Heterogeneity of DNA content and expression of cell cycle genes in axenically growing *Entamoeba histolytica* HM1:IMSS clone A.. Mol Biochem Parasitol.

[10] Das S, Lohia A (2002). Delinking of S phase and cytokinesis in the protozoan parasite *Entamoeba histolytica*.. Cell Microbiol.

[11] Byers J, Eichinger D (2005). *Entamoeba invadens*: restriction of ploidy by colonic short chain fatty acids.. Exp Parasitol.

[12] Ganguly A, Lohia A (2001). The cell cycle of *Entamoeba invadens* during vegetative growth and differentiation.. Mol Biochem Parasitol.

[13] Lohia A (2003). The cell cycle of *Entamoeba histolytica*.. Mol Cell Biochem.

[14] Das S, Mukherjee C, Sinha P, Lohia A (2005). Constitutive association of Mcm2-3-5 proteins with chromatin in *Entamoeba histolytica*.. Cell Microbiol.

[15] Lohia A, Mukherjee C, Majumder S, Dastidar PG (2007). Genome Re-duplication and Irregular Segregation Occur During the Cell Cycle of *Entamoeba histolytica*.. Biosci Rep.

[16] Mukhopadhyay RM, Chaudhuri SK (1996). Rapid in vitro test for determination of anti-amoebic activity.. Trans R Soc Trop Med Hyg.

[17] Ali IK, Mondal U, Roy S, Haque R, Petri WA (2007). Evidence for a link between parasite genotype and outcome of infection with *Entamoeba histolytica*.. J Clin Microbiol.

[18] Clark CG, Diamond LS (2002). Methods for cultivation of luminal parasitic protists of clinical importance.. Clin Microbiol Rev.

[19] Sanchez LB, Enea V, Eichinger D (1994). Increased levels of polyadenylated histone H2B mRNA accumulate during *Entamoeba invadens* cyst formation.. Mol Biochem Parasitol.

[20] Gonzalez J, Bai G, Frevert U, Corey EJ, Eichinger D (1999). Proteasome-dependent cyst formation and stage-specific ubiquitin mRNA accumulation in *Entamoeba invadens*.. Eur J Biochem.

[21] Banerjee S, Das S, Lohia A (2002). Eukaryotic checkpoints are absent in the cell division cycle of *Entamoeba histolytica*.. J Biosci.

[22] Dvorak JA, Kobayashi S, Alling DW, Hallahan CW (1995). Elucidation of the DNA synthetic cycle of *Entamoeba* spp. using flow cytometry and mathematical modeling.. J Eukaryot Microbiol.

[23] Bhattacharya S, Som I, Bhattacharya A (1998). The ribosomal DNA plasmids of *Entamoeba*.. Parasitol Today.

[24] Sharpe PT, Knight GM, Watts DJ (1984). Changes in the DNA content of amoebae of *Dictyostelium discoideum* during growth and development.. Biochem J.

[25] Kayman SC, Reichel M, Clarke M (1982). Motility mutants of *Dictyostelium discoideum*.. J Cell Biol.

[26] Waddell DR, Duffy K, Vogel G (1987). Cytokinesis is defective in *Dictyostelium* mutants with altered phagocytic recognition, adhesion, and vegetative cell cohesion properties.. J Cell Biol.

[27] Ehrenkaufer GM, Haque R, Hackney JA, Eichinger DJ, Singh U (2007). Identification of developmentally regulated genes in *Entamoeba histolytica*: insights into mechanisms of stage conversion in a protozoan parasite.. Cell Microbiol.

[28] Phillips BP (1973). *Entamoeba histolytica*: concurrent irreversible loss of infectivity-pathogenicity and encystment potential after prolonged maintenance in axenic culture in vitro.. Exp Parasitol.

