# Supplementary material for: Entamoeba Shows Reversible Variation in Ploidy under Different Growth Conditions and between Life Cycle Phases
Source: PLoS Negl Trop Dis. 2008 Aug 20;2(8):e281. doi: 10.1371/journal.pntd.0000281 (PMC2500184; doi:10.1371/journal.pntd.0000281)
Supplement: Table S1 — (0.05 MB DOC) [file pntd.0000281.s001.doc]

Supplementary Table S1

| Target  (*E. histolytica*) | Contig number | Primer names | Primer sequences (5’-3’) |
| --- | --- | --- | --- |
| *Actin* | 318378 | Eh Actin-s  Eh Actin-as | TGGGACGATATGGAAAAGAT  ATAGCTGGGGTGTTGAATGT |
| *Formin1* | 317142 | Eh Formin1-s  Eh Formin1-as | AGGCACAAAGAGAAAAAGAAG  AACCCACCAACATTAGCAG |
| *Ino1* | 317132 | Eh Ino1-s  Eh Ino1-as | AGAAGAAGAAGTATCACCATCAAC  ATCTCCCATAACAGCACCTC |
| *Cdk2* | 318439 | Eh Cdk2-s  Eh Cdk2-as | ACACCGAAATGACAGAAAAA  CAGCTTTACAAACTTCACCA |
| *Cyclin2* | 318346 | Eh Cyclin2-s  Eh Cyclin2-as | TCATTAGACCAACTCCTTTAGAC  TGCTAACTCAATCAAATAACGA |
| *Klp5* | 318122 | Eh Klp5-s  Eh Klp5-as | AAGAAGAACAGCAGCAACAG  TGGAACAACAAAATCACCTT |
| *Cdc6* | 316942 | Eh Cdc6-s  Eh Cdc6-as | TGATGATGAAATTGTTGGAAG  TTCCCTGTTCCTGGTGTT |
| *rDNA* | 317057 | Eh rDNA-s  Eh rDNA-as | GATTGGAATTATTTGTTTTG  ATCCTAGAATTTCACCTCTT |
|  | Accession number |  |  |
| *tRNA (AL)* | BK005648 | Eh tRNA-s  Eh tRNA-as | GATGTAGCTCAGATGGTAGA  AAGTGAAAAGGGGAATAGTA |
| *tRNA (DA)** | BK005649 | D-A5  D-A3 | CTGGTTAGTATCTTCGCCTGT  GCTACACCCCCATTAACAAT |
| *tRNA (SD)** | BK005652 | S^TGA^-D5  S^TGA^-D3 | CTCTGGATGCGTAGGTTCAA  GTATCTTCGCCTGTCACGTG |
| *tRNA (NK2)* #* | BK005650 | N-K5  N-K3 | CGAACGGCTGTTAACCGTTA  TTCCTAGCTCAGTCGGTAGA |
| *tRNA (RR)* #* | BK005651 | R-R5  R-R3 | AGCATCAGCCTTCTAAGCTG  CTTCCGACTGAGCTAACAAG |
| *tRNA (TX)*  *(Eh MRS2)✓* | BK005670AF487684 | Eh MRS2-s  Eh MRS2-as | TACCTCATACCATATCTTCG  TATCATCGTAGACAGGTTTT |
| *tRNA (YE )*  *Eh ARS1✓* | BK005661 M55340 | Eh ARS1-s  Eh ARS1-as | CGTGGTAAAAGAGAAGTAAA  GTATGTTACCAACCTACACC |

* tRNA primer sequences are taken from Ali *et al*. (2005), J. of Clinical Microbiology, 43: 5842-5847. The other primers have been designed in this study.

# tRNA primers also used for *E. invadens* semi qPCR.

✓ Earlier, these sequences were characterized as repetitive DNA sequences with different properties [Banerjee and Lohia (2002), Mol. Biol. Parasitol., 126: 35-42 and Lohia *et al*. (1990), Gene, 96: 197-203]. Later, Eh MRS2 and Eh ARS1 sequences were identified as part of tRNA sequences (Ali et al, 2005, J. of Clinical Microbiology, 43: 5842-5847). Therefore, these have been grouped as tRNA sequences.
