# Supplementary material for: Entamoeba Shows Reversible Variation in Ploidy under Different Growth Conditions and between Life Cycle Phases
Source: PLoS Negl Trop Dis. 2008 Aug 20;2(8):e281. doi: 10.1371/journal.pntd.0000281 (PMC2500184; doi:10.1371/journal.pntd.0000281)
Supplement: Table S2 — (0.04 MB DOC) [file pntd.0000281.s002.doc]

| Target  (*E. invadens*) | Locus number | Primer name | Primer sequence (5’-3’) |
| --- | --- | --- | --- |
| *β Tubulin* | EIN_157470 | Ei βTubulin-s  Ei βTubulin-as | AGCGTTGTATAAAATCACAT  GGAATCTCAGTGAACAAGTA |
| *Diaphanous* | EIN_235510 | Ei Dia-s  Ei Dia-as | AAGAGAAAGGAGAGAGAAGA  TCTTAACTTTGACGACAACT |
| *Cdk2* | EIN_110650 | Ei Cdk2-s  Ei Cdk2-as | AACAAGAGAGAGAGGAAGAC  TACAGAAATTTATCCTCCAA |
| *CyclinC* | EIN_186630 | Ei CyclinC-s  Ei CyclinC-as | CAAAAGAAATGCTGAGATAC  AATCTCACTGCAAGAAAATA |
| *Rho_GTPase* | EIN_141060 | Ei Rho-s  Ei Rho-as | CCCAGATGAATATATACCAA  ACTATGTTGTCGAGTGATGT |
| *Ubiquitin ligase E3 subunit(E3)* | EIN_058570 | Ei E3-s  Ei E3-as | ATGTATTGTCGTTTTACACC  AAATAACATGTTCAACCAAC |
| *Encystation complex component (ECC)* | EIN_058740 | Ei ECC-s  Ei ECC-as | GAGCAAAGTATCAAAGAAAA  GTCGAAAGAAGCAATAGTAA |
| *Mcm9* | EIN_060570 | Ei Mcm9-s  Ei Mcm9-as | CAGAGTGAGAAAAGTGAAAG  TTCAGTACTCTTGTGGAGAC |
| *Kinesin* | EIN_149420 | Ei Kin-s  Ei Kin-as | ACTTGTTGTGTGAAGAGAAG  TACAGAAGTCGTTATTTCGT |

Supplementary Table S2

The sequences for the above loci are available from [www.pathema.jcvi.org](http://www.pathema.jcvi.org/)
